# Amyloid Peptide Toxicity in an Animal Model of Alzheimer Disease

**DOI:** 10.1371/journal.pbio.0050313

**Published:** 2007-10-30

**Authors:** Liza Gross

Acting as motors, pumps, switches, catalysts, and in numerous other roles, proteins perform nearly all the functions of a cell. This staggering functional diversity arises from linking just 20 amino acids in seemingly endless permutations to form polypeptide chains. The sequence of amino acids in the polypeptide chain determines a protein's precise 3-D structure and biological properties. Cells have evolved multiple mechanisms to ensure proper folding, but a number of molecular and biophysical events—such as changes in pH or temperature, mutations, and oxidation—can disrupt a protein's native shape.

When polypeptides fail to achieve or maintain their proper conformation, they commonly aggregate into abnormal “amyloid” structures, which often appear as long, straight, unbranched threads—or fibrils—consisting of supercoiled “protofilaments.” Amyloid fibrils define a diverse group of degenerative conditions, including amyotrophic lateral sclerosis, prion diseases, and Alzheimer and Parkinson diseases. In Alzheimer disease, the amyloid fibrils are deposited extracellularly; however, in Parkinson and Huntington disease, similar amyloid fibrils accumulate in the cytoplasm and nucleus of the cell respectively. How amyloid formation promotes disease has generated considerable debate, though mounting evidence implicates the early protofibrillar aggregates as the toxic species.

In a new study, Leila Luheshi et al. worked with the fruit fly *Drosophila* to identify the intrinsic determinants of amyloid β (Aβ) pathogenicity in an animal model of Alzheimer disease. (Aβ peptide is a primary component of amyloid plaques in the brains of patients with Alzheimer disease.) Determining how amyloid formation causes disease requires a better understanding of the molecular and biophysical conditions that promote protein aggregation. But such an understanding has proven technically challenging, in part because protein misfolding and aggregation in test tubes can't replicate cellular pathways designed to mitigate the toxic effects of these events. Luheshi et al. circumvented this problem by integrating computational predictions of protein aggregation propensities with in vitro experiments to test the predictions and in vivo mutagenesis experiments to link predicted aggregation propensity with observed neurodegeneration in the flies.

Aβ peptides have between 39 and 43 amino acid sequences (called isoforms), indicated as Aβ_39_, Aβ_40_, and so on. Expression of Aβ_42_ in the *Drosophila* central nervous system produces neuron-damaging deposits and leads to abnormal locomotor behavior and shortened life span, as well as learning and memory problems. The researchers computed the aggregation propensities for each of Aβ_42_'s 798 possible single amino acid substitutions—based on the physical and chemical properties of the amino acids—along with those of the pathogenic variant E22G Aβ_42_, which is associated with the inherited form of Alzheimer disease.

They identified 17 Aβ mutational variants with wide-ranging aggregation propensities, expressed them in the *Drosophila* central nervous system, and then measured the variants' effects on locomotion and longevity. Fly lines expressing the form of the Aβ peptide that has a low aggregation propensity and is associated with health in humans (Aβ_40_) had a life span that was equivalent to healthy flies. Those expressing the nonmutant (wild-type) Aβ_42_ peptide, which deposits rapidly in the human brain, died sooner; but worse still were the flies expressing the highly aggregatory E22G peptide. A few variant lines (F20E and 131E/E22G) showed better survival and mobility than the wild-type flies (as determined by the number of flies reaching the top of a pipette after 45 seconds), while other variants shortened the flies' life spans.

**Figure:**
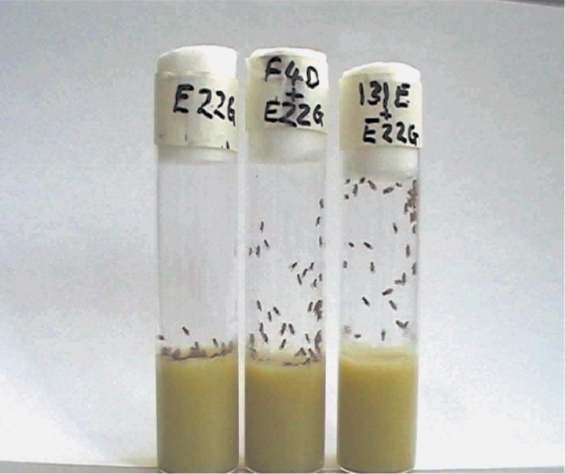
The locomotor ability of flies expressing the pathogenic Aβ peptide variant, which is associated with Alzheimer disease, is severely compromised compared with that of flies carrying less-harmful Aβ peptide variants.

Overall, the researchers found a clear correlation between a variant's predicted tendency to aggregate and its influence on fly longevity. The same relationship was seen between predicted aggregation propensity and locomotion (for a representative sample of variant lines), though a few variants did not follow this pattern. To understand why, the researchers ran additional experiments on two peptides—one whose neuronal effects matched its predicted aggregation propensity (F20E) and one whose didn't (131E/E22G).

As predicted, F20E reduced the Aβ_42_ peptide's tendency to aggregate. The F20E Aβ_42_ peptide aggregated much more slowly than the wild-type Aβ_42_ did in test tubes; and where the variant left no deposits in fly brains after 20 days, the wild-type peptide left extensive deposits. Since the F20E peptide could form amyloid fibrils in the test tube (even though it did so slowly) but didn't leave deposits in the brain, the researchers concluded that the F20E mutation inhibited aggregation enough for cells to dispose of the aberrant peptide—thus accounting for the flies' enhanced survival and behavior.

The 131E/E22G peptide, however, showed no correlation between predicted aggregation propensity—expected to resemble that of the pathogenic E22G peptide—and its increased survival and mobility. Nonetheless, the peptide aggregated at rates similar to the Alzheimer variant in vitro as well as in the fly brains. But because the 131E/E22G peptide deposits were not accompanied by cavities in brain tissue—a telltale sign of neurodegeneration—the flies showed no neurological deficits.

This finding fits with reports that the density of Aβ plaques in elderly patients with Alzheimer disease does not correlate with the severity of clinical symptoms—and that the soluble protofibrillar aggregates, not the mature amyloid plaques, cause neurodegeneration. Recomputing the propensities of each Aβ variant to form these protofibrillar species revealed not only an improved overall correlation with toxicity but it also brought the previously anomalous 131E/E22G variant in line with the prediction algorithm.

Altogether, these results show that Aβ's toxic effects in a living organism can be predicted based on a computational analysis of its tendency to form protofibrillar aggregates. And even though cells have evolved multiple mechanisms to regulate folding, the researchers argue, it is the intrinsic tendency of the peptide's sequence to aggregate that governs its pathological propensity. Though the researchers focused on the peptide most closely associated with Alzheimer disease, they believe their approach will work for many other diseases as well.

